# Diagnostic laparoscopy and oophorectomy for ovarian vein thrombosis in a patient with COVID-19: a surgical case report and literature review

**DOI:** 10.1093/jscr/rjab389

**Published:** 2021-09-08

**Authors:** Rebecca Glanzer, Nicole Rogers, Ryan J Patrick, Jeanne Hassebroek-Johnson

**Affiliations:** Sanford School of Medicine, Vermillion, SD 57069, USA; Sanford School of Medicine, Vermillion, SD 57069, USA; Sanford School of Medicine, Vermillion, SD 57069, USA; Department of Obstetrics and Gynecology, Sanford Health, Sioux Falls, SD 57117, USA

## Abstract

Ovarian vein thrombosis (OVT) is a rare condition most frequently associated with pelvic inflammatory disease (PID), malignancy or the immediate postpartum period. This case study reports on a 56-year-old woman who developed OVT 11 days after a positive COVID-19 diagnosis. Imaging including abdominal/pelvic computed tomography, transvaginal Doppler ultrasound and transabdominal pelvic ultrasound failed to definitively diagnose the thrombotic etiology of the patient’s presentation. Ultimately, laparoscopic visualization and subsequent oophorectomy were necessary for diagnostic and therapeutic purposes. The patient did not have underlying malignancy, recent surgical history, history of PID or any history of previous thromboembolic events. Therefore, this report contributes further evidence to the growing knowledge of systemic manifestations associated with COVID-19 that may require surgical intervention.

## INTRODUCTION

Ovarian vein thrombosis (OVT) is a rare condition, often seen in the postpartum period, and is frequently associated with pelvic inflammatory disease, malignancy, inflammatory bowel disease, thrombophilia, and pelvic and gynecologic surgeries [1–3]. It affects ~0.05–0.16% of pregnancies in the postpartum period [2, 4]. The classic triad of presentation includes pelvic pain, fever and right-sided abdominal mass, but it can be associated with tachycardia, hypotension, tachypnea, lower quadrant or flank pain, nausea, vomiting, malaise, shortness of breath and pyuria [2, 4]. A variety of imaging modalities can be used for diagnosis, and treatment is generally anticoagulation.

## CASE REPORT

The patient is a 56-year-old woman who presented to the emergency room with a chief complaint of a 6-hour history of worsening ‘cramping, burning’, right lower quadrant abdominal pain aggravated by movement with occasional radiations to the low back. She tested positive for COVID-19 11 days prior. The patient reported mild residual nausea and diarrhea but denied other associated symptoms. Past medical history included restless leg syndrome and hypertension. Past surgical history was significant for laparoscopic hysterectomy 3 years prior and ovarian cystectomy in her teenage years. The patient had three previous vaginal deliveries, and her last menstrual period was 7 years prior. Vitals signs were stable in the emergency department. Physical examination demonstrated isolated abdominal tenderness in the right lower quadrant with no distension or rebound tenderness. Pertinent labs included mild hypokalemia and mild thrombocytopenia. An abdominal and pelvic computed tomography (CT) with contrast showed inflammatory changes and fluid along the right gonadal vein with no definite thrombosis; the right ovary was not distinctly identified. A transvaginal Doppler ultrasound revealed appropriate arterial flow with questionable visualization of the right ovary, no visualization of the left ovary and a small amount of free fluid in the pelvis. A transabdominal pelvic ultrasound was able to identify a relatively enlarged right ovary with irregular borders and a normal left ovary. Considering the patient’s clinical presentation and high index of suspicion for ovarian torsion, surgical intervention was recommended, and the patient consented for diagnostic laparoscopy.

Intraoperatively, surgical access to the abdomen was obtained and an initial survey of the pelvis demonstrated surgically absent uterus and bilateral fallopian tubes, normal appearing left ovary and a necrotic, indurated right ovary without evidence of torsion. The distal right ovarian vessels and surrounding local tissues appeared indurated with evidence of thrombus ~2-cm cephalad to the ovary extending down to the ovary. These findings suggested an ongoing thrombotic process localized to the ovary as there was no evidence of active infection or involvement of neighboring structures. The area of induration correlated with previously noted findings on the CT scan. A bipolar cautery and cutting device was used to dissect through the mesovarium and infundibulopelvic ligament. Once the thrombus was identified, care was taken to dissect above and excise the thrombus along with the ovary. The specimen was sent for pathologic analysis, which demonstrated a blood clot within a dilated vessel. Postoperatively, the patient reported resolution of her abdominal pain, tolerated oral intake well and was started on heparin. Her hospital course was uneventful, lasting 2 days. The patient was discharged on an anticoagulation regimen of 5 mg apixaban BID tapered to 2.5 mg BID after 6 months. Six months postoperatively, the patient was doing well without recurrence or need for further surgical intervention.

## DISCUSSION

Reports of OVT in patients infected with SARS-CoV-2 have been previously described in one pregnant, one postpartum and three postmenopausal patients [3, 5–7]. A hypercoagulable state associated with COVID-19 has come to the forefront in recent research, with many pathophysiologic mechanisms proposed. One mechanism theorizes SARS-CoV-2 infects angiotensin-converting enzyme 2 (ACE2) receptors, present on, among other tissues, endothelial cells, leading to cellular injury and endothelitis [3, 7]. Another suggests that patients with severe COVID-19 infection are exposed to high concentrations of cytokines such as IL2, IL7, IL10, GCSF, IP10, MCP1, MIP1A and TNF-a, which can lead to severe inflammatory responses and activation of the coagulation cascade [5, 7]. Historically, patient characteristics correlated with higher ovarian thrombosis risk include those with respiratory failure, cardiovascular failure, obesity, history of previous thrombosis, >3 days of bed rest or age over 40 [5]. This patient denied a history of preexisting thromboembolic disorder, recent surgeries or history of malignancy, suggesting COVID-19 as a likely mechanism for her development of ovarian thrombosis.

Ultrasound, CT and magnetic resonance imaging are commonly used imaging modalities for suspected OVT. However, these imaging modalities often produce nonspecific findings that require reliance on clinical judgment to determine when surgical intervention is necessary, as demonstrated in this case. A need for timely diagnostics and therapeutic intervention for this patient paired with a high index of suspicion guided the decision to pursue surgical exploration. Surgical exploration and subsequent right oophorectomy confirmed the thromboembolic etiology and relieved the patient’s pain. Ultimately, surgical exploration is the gold standard for definitive diagnosis of OVT. The case reported upon here reflects the importance of relying on clinical judgment rather than imaging modalities alone to guide the course of management in cases of suspected OVT. When OVT is confirmed, rigorous adherence to anticoagulation therapy is imperative for treatment, as propagation or persistence of the offending clot can predispose patients to pulmonary emboli, ovarian abscesses, ovarian infarctions, septic thrombophlebitis, uterine necrosis or ureteral compression [4, 8]. While there is no specific regimen for treating thromboembolism secondary to COVID-19 infection, the literature supports the use of apixaban or enoxaparin/acenocoumarol.

OVT is rarely observed outside of the postpartum period; however, this case represents the sixth patient reported upon this year who developed OVT following COVID-19 infection. This case report, in conjunction with the many previously published incidents of similar thromboembolic events, lends support to the growing body of literature describing the thromboembolic systemic manifestations of COVID-19. This case demonstrates the imperative role clinical examination and suspicion play in diagnosing OVT, as imaging is frequently equivocal. Surgical intervention proved vital for definitive diagnosis and treatment.
